# Enhancing Adverse Drug Event Detection in Electronic Health Records Using Molecular Structure Similarity: Application to *Pancreatitis*

**DOI:** 10.1371/journal.pone.0041471

**Published:** 2012-07-24

**Authors:** Santiago Vilar, Rave Harpaz, Lourdes Santana, Eugenio Uriarte, Carol Friedman

**Affiliations:** 1 Department of Biomedical Informatics, Columbia University Medical Center, New York, New York, United States of America; 2 Department of Organic Chemistry, Faculty of Pharmacy, University of Santiago de Compostela, Santiago de Compostela, Spain; Indian Institute of Toxicology Reserach, India

## Abstract

**Background:**

Adverse drug events (ADEs) detection and assessment is at the center of pharmacovigilance. Data mining of systems, such as FDA’s Adverse Event Reporting System (AERS) and more recently, Electronic Health Records (EHRs), can aid in the automatic detection and analysis of ADEs. Although different data mining approaches have been shown to be valuable, it is still crucial to improve the quality of the generated signals.

**Objective:**

To leverage structural similarity by developing molecular fingerprint-based models (MFBMs) to strengthen ADE signals generated from EHR data.

**Methods:**

A reference standard of drugs known to be causally associated with the adverse event *pancreatitis* was used to create a MFBM. Electronic Health Records (EHRs) from the New York Presbyterian Hospital were mined to generate structured data. *Disproportionality Analysis* (DPA) was applied to the data, and 278 possible signals related to the ADE *pancreatitis* were detected. Candidate drugs associated with these signals were then assessed using the MFBM to find the most promising candidates based on structural similarity.

**Results:**

The use of MFBM as a means to strengthen or prioritize signals generated from the EHR significantly improved the detection accuracy of ADEs related to *pancreatitis*. MFBM also highlights the etiology of the ADE by identifying structurally similar drugs, which could follow a similar mechanism of action.

**Conclusion:**

The method proposed in this paper provides evidence of being a promising adjunct to existing automated ADE detection and analysis approaches.

## Introduction

The main objective of pharmacovigilance involves the collection, monitoring, assessment and evaluation of adverse effects of medications and other biological products from healthcare providers and patients. There are different Spontaneous Reporting System (SRS) databases, such as the FDA’s Adverse Event Reporting System (AERS) [1], the European Medicines Agency (EMA) [2] and the World Health Organization (WHO) international database [3] that have been designed to collect reports of suspected adverse drug events (ADEs) for these purposes. Despite their success and strengths they have some limitations [4]. As an example, the number of patients at risk who are taking a drug cannot be determined, adverse reactions are underreported, and reporting is biased. Clinical information in Electronic Health Records (EHRs) has emerged as a new source that can provide important information to complement and improve drug safety surveillance strategies [5], [6]. It is believed that the integration of diverse information sources can lead to an improved surveillance system [7], [8].

Different data mining algorithms (DMAs) [5], [9], based on *disproportionality analysis* (DPA) have been developed to assist in identifying safety signals in pharmacovigilance databases of potentially novel ADEs that merit further investigation. However, signals generated through DMAs based on EHR data also have some limitations and challenges that are different from those associated with SRS data. EHR data consists of information associated with the process of care, which is not focused on the reporting of adverse events, and therefore ADEs are often sparse in EHR data and occur much less frequently than other types of clinical information, such as treatments, disorders, and symptoms. In addition, drug-ADE relations are typically not expressed explicitly in EHRs, and DMA methods based on co-occurrence in a report are used to find associations from a broad variety of information in the patient reports, such as treatment indications, co-morbidities, other symptoms or medications. Therefore, the statistically-based associations between a drug and an adverse event (AE) represent drug-AE relations that are not necessarily ADEs, such as treatment relations. Since use of the EHR for pharmacovigilance is a relatively new area of research, it would be useful to develop and evaluate new methods to enhance the accuracy of signals generated through EHR-based DMAs.

In previous work, we demonstrated that molecular similarity analysis is a valuable tool to improve the accuracy DPA based ADE detection in AERS [10]. Exploiting the premise widely accepted in medicinal chemistry that similar molecules can have similar biological properties [11], the drugs determined by the model as being structurally similar to an ADE reference standard set, could cause the same ADE following a similar pharmacological mechanism. In this article, we showed that the application of MFBM can be easily extended to ADE detection based on EHR data. Our results demonstrate that the integration of both methodologies facilitates the detection of the ADE *pancreatitis*.

## Materials and Methods

### Ethics Statement

New York Presbyterian Hospital Electronic Health Records were analyzed after obtaining IRB approval from Columbia University Medical Center committee (consent was given through waiver of authorization; protocol number: IRB-AAAD6669).

### Materials

#### EHR data

The EHR data included approximately 1.2 million narrative patient notes from 2004 to 2010. Admission notes, discharge summaries and outpatient visits for a total of approximately 178,000 patients at New York Presbyterian Hospital were analyzed after obtaining IRB approval.

#### Reference standard dataset

A reference standard of 253 drugs reported to cause the ADE *pancreatitis* was collected. Information in the reference standard was compiled from Micromedex/Drugdex, review articles, case reports, and reliable websites (the complete dataset and the references are given in Table S1). Drugs following two inclusion criteria were taken into account in the reference standard: 1) well-established by Micromedex (a trusted medical database), or by literature reviews or reports where the *pancreatitis* follows a reasonable temporal sequence from administration of the drug and the ADE is confirmed by de-challenge/re-challenge (cessation of the drug and the symptoms and new exposure to the drug and reappearance of the symptoms); 2) probable/possible where the drug is considered be responsible for the adverse effect but other possible causes are not totally excluded (more information is provided in Table S1).

#### Drug structure

The DrugBank database [12] was used to obtain the structures of drugs included in this study. Some drugs whose structures were not available in DrugBank were manually represented using the Molecular Operating Environment (MOE) software [13].

### Methods

**Figure 1:**
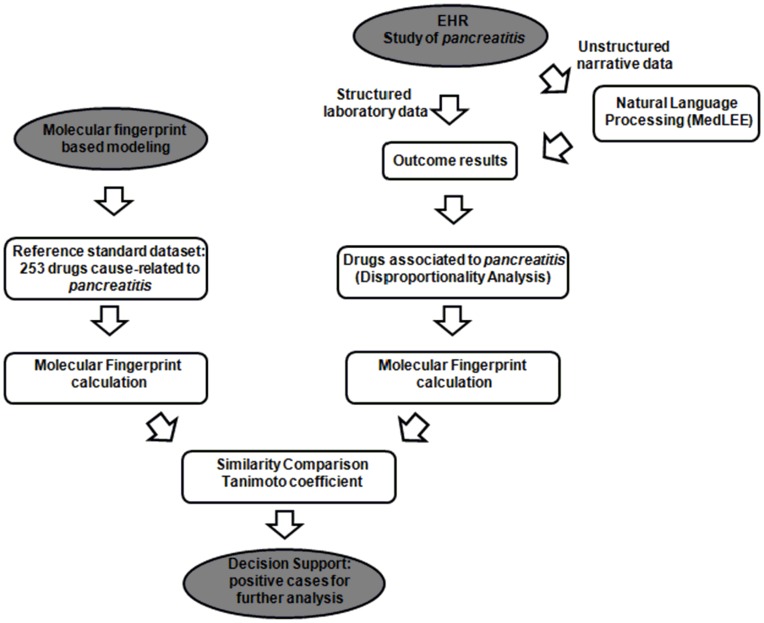
Flowchart of the ADE detection process for *pancreatitis*.

An overview of the different steps is shown in Figure 1, and a more detailed description is provided below: first, unstructured narrative and structured laboratory data from the EHR is processed to generate a set of candidate-drugs associated with *pancreatitis* using DPA. The molecular fingerprints are computed and the similarity of structures of the candidate-drugs are compared to the structures in our reference standard set through MFBM providing more evidence of a possible novel ADE that would be of interest to study further.

#### EHR data mining

The natural language processing (NLP) system MedLEE [14] was used to process the unstructured narrative data, and to extract and normalize relevant clinical entities, such as medications, diseases, symptoms, and associated temporal information. In addition, structured data was also obtained, consisting of abnormal laboratory test results that were associated with *pancreatitis.* Determining the *pancreatitis* outcome was based on laboratory test values [15], [16], which were extracted from structured EHR data (amylase ≥300 U/L or lipase ≥120 U/L), as these values are standardly used to determine potential occurrences of *pancreatitis*. Drug names were standardized and mapped to UMLS codes using MedLEE [14], and then generic names were obtained using RXNORM [17]. As an example, the brand name *Videx* was extracted from a note and then mapped to the UMLS code C0592249 by MedLEE, and subsequently RXNORM was used to map it to the generic name *didanosine* (UMLS code C0012133). A detailed process has been described in previous publications [18], [19]. The standard DPA method using the Odds Ratio (OR) measure was used to generate signals consisting of statistical associations between drugs and the event *pancreatitis* [9], [20]. DPA is based on frequency analysis consisting of 2×2 contingency tables containing drug-event pairs. OR is a measure of association between drugs and adverse events that can be calculated from the contingency table [9], [20]. Associations were quantified by the lower 5^th^ percentile of the Odds Ratio measure (OR05) [9]. To qualify as signals, associations had to meet two criteria: (1) OR05>1.25; (2) associations must pass a statistical test of independence P-value <0.05, based on the one-sided Fisher exact test and a Bonferroni correction, a conservative adjustment for multiple comparisons.

#### Molecular fingerprint-based modeling (MFBM)

The development process of this type of MFBM has been described in more detail in our previous publication [10]. The structures of the drugs involved in this study (i.e., the drugs in the reference standard and the candidate drugs generated by DPA method) were downloaded from the DrugBank database [12] and subjected to different preprocessing steps with the module Wash in the Molecular Operating Environment (MOE) software [13]. In this module, simple metal salts were disconnected and only the active ingredient was retained (i.e. the largest molecular fragment), the protonation state was considered neutral and explicit hydrogen atoms were added.

In the next step, four different molecular fingerprints were calculated for the drugs using the software MOE to carry out a comparative study of their performances: a) MACCS (MACCS structural keys), b) TGD (Typed Graph Distances), c) TGT (Typed Graph Triangles) and d) GpiDAPH3 (Graph 3-Point Pharmacophore) [13]. The basic idea is to represent a molecule using a bit vector that codifies the existence or absence of structural features, functional groups, pharmacophore features or molecular properties [21]–[25]. All the molecular fingerprints were calculated from the 2D molecular graph. MACCS fingerprints codify 166 structural keys. As an example, using MACCS fingerprints, some substructures represented in the molecule C_7_H_13_-NH_2_ are: bit 19-seven membered ring, bit 84-NH_2_ (amine group). TGD is a 2-point pharmacophore fingerprint codifying pair of atoms using graph distance and two atom types (the atom type could be donor, acceptor, polar, anion, cation, hydrophobe). TGT is a similar fingerprint but codifies triplets of atoms (3-point pharmacophore). GpiDAPH3 also codifies 3 pharmacophoric features calculated from the 2D molecular graph. All triplets of atoms are coded using graph distances and atom types (there are 8 possible atom types).

The final step consisted of similarity assessments between fingerprints of pairs of drugs using the Tanimoto coefficient (TC) measure of similarity. TC, also known as the Jaccard index, is one of the measurements most widely applied in the scientific literature for measuring similarity [24]. The range for the TC covers values from 0 to 1, where 0 means “minimum similarity” and 1 means “maximum similarity”. The TC between two fingerprints A and B is defined as:
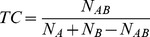
where N_A_ is the number of features present in fingerprint A, N_B_ is the number of features present in fingerprint B, and N_AB_ is the number of features present in both fingerprints A and B.

To compare a test set of drugs against the reference standard set of drugs known to cause *pancreatitis*, a similarity matrix file based on TC for all the drugs was calculated using the Fingerprint Cluster module and the sim_matrix2txt.svl script in MOE [13]. Therefore Sim_ij_ is the similarity score (TC values) between the i*th* drug and the j*th* drug in the matrix. The final similarity scoring provided by the model for a drug in the test is defined as the maximum pairwise TC obtained against each drug in the *pancreatitis* reference standard set. As an example, the drug *entecavir* in the test set was compared through TC to all the drugs in the reference standard and the maximum TC was considered the final score pointing out that the most similar drug in the reference standard was *ganciclovir*.

#### Evaluation

Performance evaluation of the method using DPA by itself [9] was compared to the method that combined DPA with MFBM. The evaluation centered on the proportion of true signals identified by each of the approaches based on the reference standard.

#### DPA by itself

The evaluation of DPA by itself was based on comparing the drugs that were selected with OR05 value greater than the previously established cutoff of 1.25 with the drugs in the *pancreatitis* reference standard dataset. Since only associations above the estimated cut-off were taken into account, the ROC curve cannot be plotted with all the drugs included in the EHR. For this reason the precision of the method (TP/TP+FP) was calculated as a standard comparative measurement of the performance. The method was compared to random results through one-sided Fisher’s exact test using the DrugBank database [12] as a resource, as explained below in Results.

#### Combination of DPA and MFBM

All the drug candidates highlighted using DPA were subjected to MBFM-based analysis. As described previously, a comparison between molecular fingerprints from candidates selected by OR05 and the reference standard set of drugs was carried out through the calculation of the Tanimoto coefficient (TC). The maximum pairwise TC obtained against each drug in the *pancreatitis* reference standard set was considered to be the final similarity score.

The performance of the MFBM was assessed through a leave-one-out cross validation method. Each drug included in both sets (candidates selected through OR05 and already included in the reference standard set) was taken out and evaluated by the model in order to compare the performance with the rest of the candidate drugs selected by OR05. Precision-Recall and Receiver Operating Characteristic (ROC) curves were plotted, considering as true positives the drugs included in our reference standard and false positives the rest of candidate drugs.

When evaluating performance, the false positives according to our system are drugs not included in our reference standard. Nevertheless, it is possible that some of the drugs selected and not included in the reference standard are causally related to *pancreatitis*. For this reason, a bibliographic search using Micromedex/Drugdex database [26], [27] and case reports from the literature was carried out to confirm whether the drugs found by the method but not included in the reference standard dataset could be the cause of the adverse event under study (see Table S2). A final evaluation of the combined model has been completed for this test set through Precision-Recall and ROC curves.

## Results

### Performance of DPA

**Figure 2:**
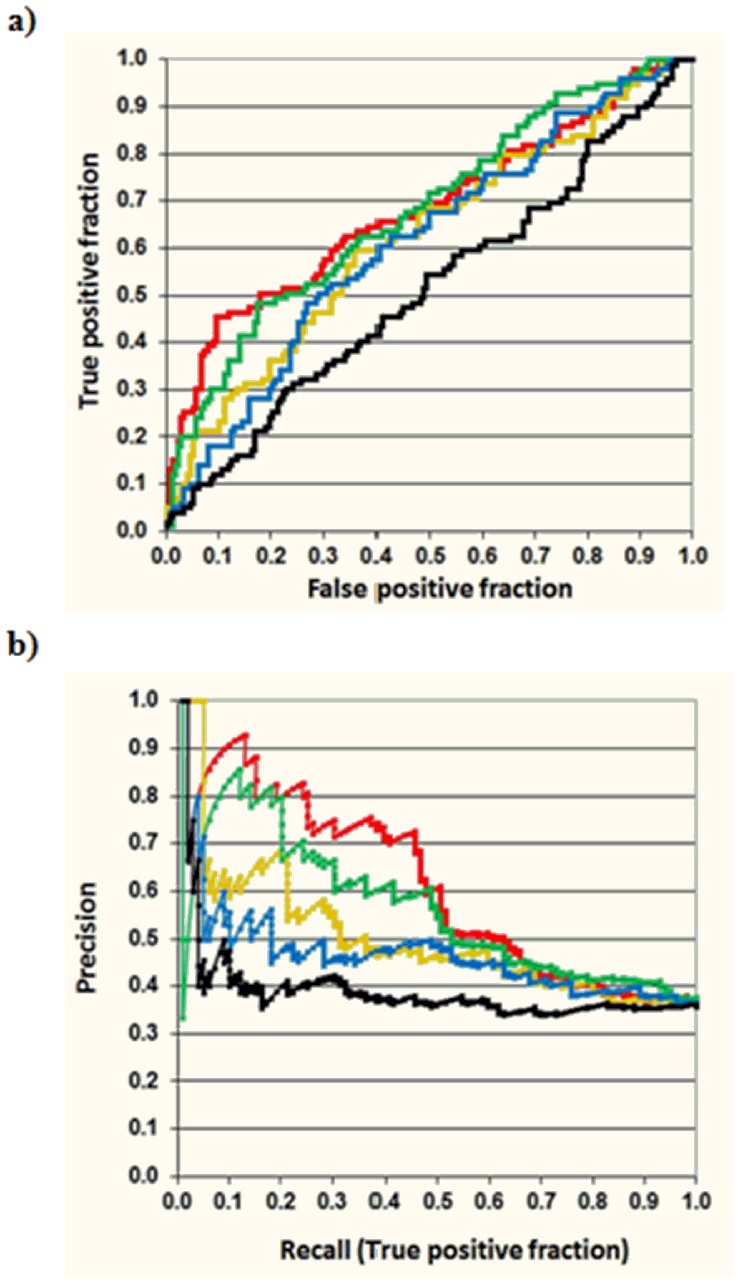
Receiver Operating Characteristic (ROC) (a) and Precision-Recall (b) curves evaluating the set of 278 EHR ADE candidates with OR05 and different MFBMs. It is worth noting that although OR05 algorithm is very useful to originate the first set of 278 candidate drugs related to *pancreatitis* (99 out of 278 drugs were already included in the *pancreatitis* reference standard set), the precision of the method is constant within this set. However, an improvement of the precision in top positions can be achieved using MFBM (in the graphic: black-OR05, red-MACCS, green-GpiDAPH3, yellow-TGT, blue-TGD).

The EHR from New York Presbyterian Hospital was mined looking for associations between drugs and the adverse event *pancreatitis*, and 278 drugs were found to be associated with the ADE using the DPA method by itself. Of those, 99 drugs were already included in the *pancreatitis* reference standard dataset established previously (see Tables S1 and S2). The precision of the method was calculated as the ratio of true positive cases divided by all the positive cases (Precision  =  TP/(TP+FP)). The overall precision of the EHR analysis is 0.36. The method was compared to random results using the DrugBank database [12], containing 1660 approved drugs (small drugs, biotech and nutraceuticals). Based on expectation, if a random subset of 278 drugs in DrugBank was selected, 42 drugs included in the reference standard would be found. The estimated precision of a method that randomly selects drug candidates was 0.15. The *p*-value for the probability that *Disproportionality Analysis* (DPA) identified 99 reference standard drugs in the subset of 278 candidates is very unlikely (p<.001). Table 1 shows the performance of DPA at different top positions. These results point out the usefulness of the application of EHR in the detection of adverse events in drugs, since 99 out of 278 associations were found in the *pancreatitis* reference standard database. However, if the Precision-Recall and Receiver Operating Characteristic (ROC) curves are plotted for the 278 candidates using OR05 as the scoring function, it is possible to observe that the precision of the method barely improves in top positions (see Table 1 and Figure 2). Nevertheless, as it is explained in the next section, an improvement in ADE detection is still possible through the combination of DPA with MFBM techniques.

**Table 1:** Performance of DPA compared to DPA+MFBM (DPA combined with MACCS, GpiDAPH3, TGD and TGT molecular fingerprints) in different TOP positions.

Number of reference standard drugs in different TOP positions					
	DPA+MACCS	DPA+GpiDAPH3	DPA+TGD	DPA+TGT	DPA
**TOP-10**	9	8	5	6	4
**TOP-25**	20	20	14	16	10
**TOP-50**	37	30	23	28	20
**TOP-75**	47	44	34	36	31
**TOP-100**	52	51	48	46	38
**TOP-125**	62	60	56	59	46
**TOP-150**	66	67	63	63	55
**Precision in different TOP positions**					
	**DPA+MACCS**	**DPA+GpiDAPH3**	**DPA+TGD**	**DPA+TGT**	**DPA**
**TOP-10**	0.90	0.80	0.50	0.60	0.40
**TOP-25**	0.80	0.80	0.56	0.64	0.40
**TOP-50**	0.74	0.60	0.46	0.56	0.40
**TOP-75**	0.63	0.59	0.45	0.48	0.41
**TOP-100**	0.52	0.51	0.48	0.46	0.38
**TOP-125**	0.50	0.48	0.45	0.47	0.37
**TOP-150**	0.44	0.45	0.42	0.42	0.37
**Enrichment factor in different TOP positions**					
	**DPA+MACCS**	**DPA+GpiDAPH3**	**DPA+TGD**	**DPA+TGT**	**DPA**
**TOP-10**	2.53	2.25	1.40	1.68	1.12
**TOP-25**	2.25	2.25	1.57	1.80	1.12
**TOP-50**	2.08	1.68	1.29	1.57	1.12
**TOP-75**	1.76	1.65	1.27	1.35	1.16
**TOP-100**	1.46	1.43	1.35	1.29	1.07
**TOP-125**	1.39	1.35	1.26	1.33	1.03
**TOP-150**	1.24	1.25	1.18	1.18	1.03

### Performance Improvement by Combining DPA with MFBM

An improvement of the precision of the method in top ranking positions can be achieved through the combination of DPA with MFBM. As described in the Methods section, different fingerprints were calculated for all the drugs used in the study. The fingerprints of the set of candidate drugs selected by *Disproportionality Analysis* (DPA) were compared to the fingerprints of drugs in the *pancreatitis* reference standard dataset through the Tanimoto coefficient (TC). Drugs included in both groups (in EHR and already in the reference standard) were taken out one by one (leave-one-out method) and evaluated by the MFBM. Precision in different top positions was calculated for all the MFBMs (see Table 1). The precision of the method improves when the combined methodology is used. A two-fold enrichment factor is achieved when a subset of 50 top drug-candidates are evaluated by MACCS fingerprints (see Table 1). Within the top 50 drugs selected by MACCS, 37 drugs were estimated as true positives (TP) and 13 drugs were found false positives (FP). The probability that the method identified 37 drugs by chance is highly unlikely (p-value<.001; one-sided Fisher’s exact test). Precision-recall and ROC curves for all 278 drug candidates selected by DPA was also plotted to show the comparative performance for the different calculated fingerprints (Figure 2). It is worth noting that although the precision of DPA is constant within this set of candidates, the method is still very useful for obtaining this first set of 278 candidate drugs related to *pancreatitis* (99 out of 278 drugs were already included in the *pancreatitis* reference standard set). However, an improvement of the precision in top positions was achieved by prioritizing the candidates generated through DPA with MFBMs.

**Figure 3:**
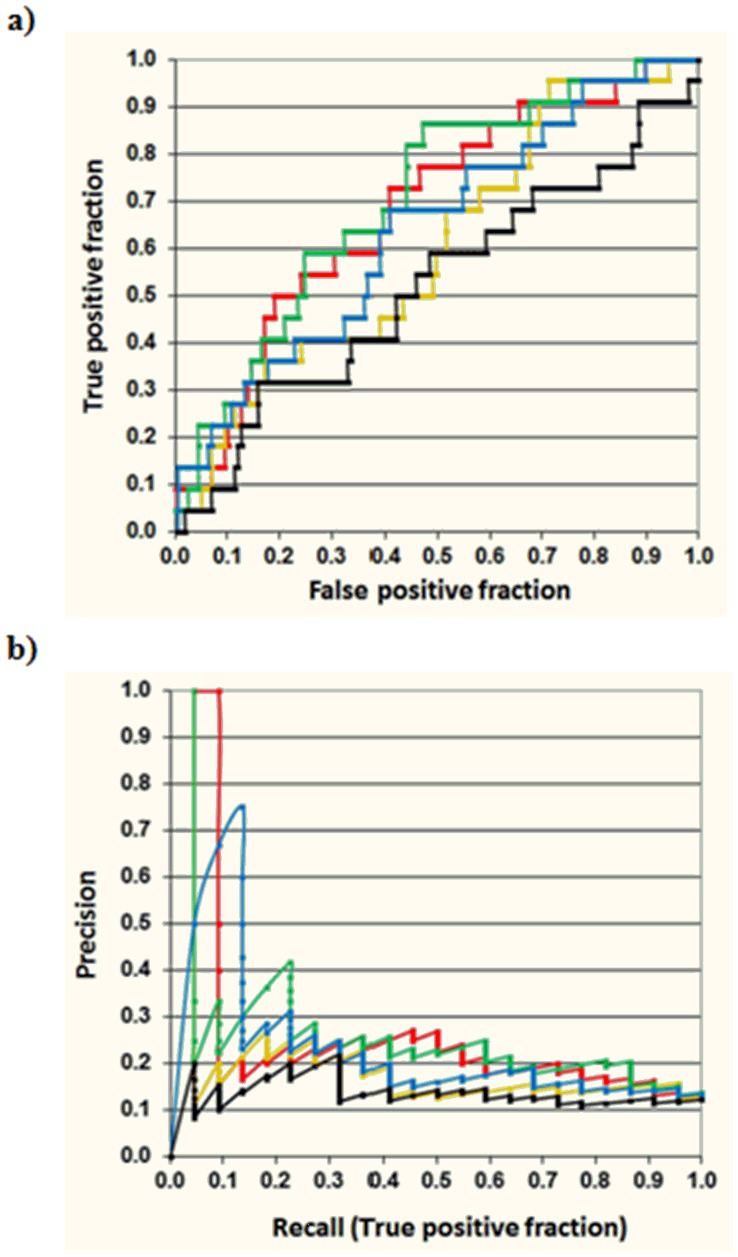
Receiver Operating Characteristic (ROC) (a) and Precision-Recall (b) curves evaluating the test set of EHR *pancreatitis* candidates (in the graphic are not included the drugs already in the reference standard: 21 true positives versus 158 false positives, black-OR05, red-MACCS, green-GpiDAPH3, yellow-TGT, blue-TGD).

A second evaluation of the combined model was completed identifying *pancreatitis* case reports in the literature and Micromedex/Drugdex database [26], [27] for the drugs selected in EHR but not included in the *pancreatitis* reference standard (see Table S2). Out of 179 drugs not included in the *pancreatitis* reference standard, 21 were considered a possible cause of *pancreatitis* and 158 were considered negative cases since no consistent information relating the drugs as the cause of the adverse event was found. Although further research will be necessary to confirm the ADE for some cases in this test set, the evaluation according to the combined method HER + MFBM provides more insights about the accuracy of the system. The overall precision in this test set is lower since the drugs included in the reference standard are not taken into account in the analysis. As shown in Figure 3, through Precision-recall and ROC plots considering this test set (21 true positives versus 158 false positives), the curves provided by MACCS and GpiDAPH3 fingerprints offer the best results in the precision improvement compared to DPA and alternative molecular fingerprint representations.

### Rationalization of the Signals Generated in EHR

The application of similarity models applied to EHR data provides a new system to rank scores of the detected associations based on the maximum similarity to a drug in the reference standard. In this manner, the drug source in the reference standard is identified by the model, for which some reports relating the drug to the adverse event were previously found. This fact facilitates the ADE evaluation process depending on the available information for the similar drug in the reference standard. The model also can help to point out different hypotheses about possible mechanisms of action. Some examples of *pancreatitis* candidate drugs not included in the reference standard identified by the combined model and with some level of evidence in the literature are described in the next section (see Table S2 for a more detailed description).

#### Examples of drugs belonging to the same pharmacological category

An example of a drug not included in the initial *pancreatitis* reference standard set is *megestrol*, a progesterone derivative with antineoplastic properties used in the treatment of carcinoma. According to our MFBM, *megestrol* is structurally similar to *norethindrone*, another progestogen drug used as oral contraceptive included in the reference standard (see Table 2 and Table S2). There are some publications that relate the drug *norethindrone* as a possible cause of *pancreatitis* with positive rechallenge [28]. Although not many reports were found relating *megestrol* as the cause of *pancreatitis*, some information is available establishing *pancreatitis* as a possible adverse event due to the use of contraceptive pills containing *megestrol* [29], [30]. However, further studies will be necessary to explain the potential adverse event in the drug.

**Table 2:** Examples of candidates selected through the combination of DPA and MFBM (MACCS fingerprints) and similar molecules in the *pancreatitis* reference standard, along with OR05 (lower 5^th^ percentile of the Odds Ratio measure of association in DPA analysis) and TC (Tanimoto coefficient) values.

EHR+MFBM drug candidate	Most similar drug in the *pancreatitis* reference standard	TC	OR05
**Same pharmacological category**			
*Megestrol*	*Norethindrone*	0.84	1.82
*Entecavir*	*Ganciclovir*	0.78	2.23
*Amphotericin B*	*Doxorubicin*	0.75	3.83
**Different pharmacological category**			
*Loperamide*	*Haloperidol*	0.79	2.60
*Pyrimethamine*	*Lamotrigine*	0.78	2.31
*Micafungin*	*Ceruletide*	0.76	3.68
*Cosyntropin*	*Caspofungin*	0.76	3.39

Different level of *pancreatitis*-causal information was found for the candidate drugs in the literature.

*Entecavir* is a nucleoside analog used in clinic as an antiviral for the treatment of hepatitis B. A possible role of *entecavir* in the development of *pancreatitis* has been discussed previously [31]. Elevations in serum amylase were also reported in patients receiving *entecavir* although no clinical *pancreatitis* was diagnosed [32]. Our system detected a structural similarity between *entecavir* and *ganciclovir*, another antiviral drug used to treat cytomegalovirus infections which was included in the reference standard because it was identified in the literature and in Micromedex causally related to *pancreatitis* [26], [33].

Another case found in the EHR associated with *pancreatitis* is the polyene antifungal antibiotic drug *amphotericin B*. This drug was also detected by MFBM as a drug similar to *doxorubicin*, an anthracycline antibiotic used in clinic in the treatment of different types of cancer (see Table 2). Although *amphotericin B* could be the treatment for fungal infections associated to *pancreatitis*, there are some reports in the literature that confirm the potential risk of this drug. A report describes an increased serum lipase levels with clinical signs of *pancreatitis* in some patients treated with liposomal *amphotericin B* therapy [34]. Another report describes a case of *pancreatitis* in an HIV patient possibly due to *amphotericin B* [35].

#### Examples of drugs belonging to different pharmacological categories

The combination of the DPA and MFBM can also detect drugs that belong to different pharmacological classes (see Table 2). An interesting example of drug pointed out by MFBM is *loperamide*, a piperidine derivative opioid-receptor agonist that is very effective for the treatment of diarrhea. The structure of *loperamide* is similar to *haloperidol*, a drug in the reference standard with different pharmacological category since *haloperidol* is a butyrophenone belonging to antipsychotic medications and used in the treatment of schizophrenia. There are some reports in the literature that confirm the potential adverse effect of *lopiramide* probably due to a spasm at the sphincter of Oddi or inhibition of the release of pancreatic polypeptide [36]–[38]. On the other hand, *haloperidol* was reported to cause *pancreatitis* in a study of antipsychotic drugs inducing *pancreatitis* [27], [39]. The temporal relationship between the adverse event and the beginning of the therapy could indicate a causal relationship.

Another example of similar drugs belonging to different pharmacological classes is *pyrimethamine* and *lamotrigine* (drug included in the reference standard). *Pyrimethamine*, an antiparasitic drug used in the treatment of *malaria* and *toxoplasmosis*, is selected by MFBM as a drug structurally related to *lamotrigine* (both drugs present a chloro substituted phenyl with a diamine azine ring), an anticonvulsant used to treat epilepsy, bipolar disorders and depression. *Pancreatitis* is described as a possible ADE for the combination pyrimethamine/sulfadoxine [40]. However, more studies will be necessary to confirm the ADE.

An interesting case to study further is *micafungin*, an antifungal drug indicated for the treatment of candidiasis by inhibiting the production of 1,3-beta-D-glucan, an important component of the fungal cell walls. Although fungi infections can occur to patients with *pancreatitis*, and therefore the prescription of an antifungal drug is necessary in some cases [41], there is also a report in the literature describing a case of acute *pancreatitis* probably caused by *micafungin* [42]. Our model detected that *micafungin* resembles the peptidic structure of *ceruletide* (see Table 2), a drug included in our reference standard and used in experimental animal models to induce *pancreatitis* [43]. Further studies will be necessary to shed some light about the relationship between *micafungin* and *pancreatitis*.

*Cosyntropin*, a derivative of adrenocorticotropic hormone used to diagnose cortisol disorders, is another case selected by DPA and MFBM as a drug related to *pancreatitis*. In this case, the information gathered in Micromedex database clearly indicates that *pancreatitis* is an ADE associated to the drug [26]. *Cosyntropin* presents some structural similarity regardless to *caspofungin*, an antifungal drug included in the reference standard set.

## Discussion

This main goal of this study is to demonstrate the usefulness of the analysis of the molecular structure to improve the precision and rationalization in the detection of drug-*pancreatitis* associations found in EHR. Although structure similarity analysis was applied to the EHR in this study, the method could be applied to other pharmacovigilance databases created to analyze postmarketing drug safety information, such as AERS, WHO or EMA. In fact, the results shown are in accordance with a previous publication analyzing the adverse event *rhabdomyolysis* from the point of view of AERS and molecular structure similarity [10].

MFBM permits the rationalization of ADE signals detected in pharmacovigilance databases through the identification of structurally similar drugs by which ADE information has been already published. This can be useful to estimate the importance of the signal generated in EHR to make decisions regarding further follow-up. The system can be used along with other pharmacovigilance methods to provide additional information and evaluate the potential relevance of the signals, such as biological and pharmacological plausibility.

The nature of the system permits the identification of drugs belonging to different pharmacological classes than the drugs included in the reference standard ADE dataset, although it is more likely the identification of drugs with similar pharmacological profiles, which can still present utility in the case of researchers not related to pharmacological backgrounds.

Although MFBM by itself can present some limitations in the detection of complex clinical adverse events due to the different biological mechanisms involved in ADEs with high molecular variability [10], the different nature regarding the databases used for pharmacovigilance, allows the combination of both methods to improve ADE detection by generating sets of drugs with enhanced enrichment factors. Since the model is based on the structural comparison against a reference standard dataset of drugs responsible for the ADE under study, the results offered by the models are directly dependent on the quality of the reference standard. This dataset should contain a heterogeneous representation of the different structural drug classes related to the ADE and be as complete as possible.

Molecular fingerprints have been widely validated in previous publications and it has been shown that they are very useful to recognize similar molecules in large databases [21]–[25]. However, some molecular fingerprints could recognize as similar two structurally different molecules when they present the same substructural or pharmacophoric features differently reorganized at molecular level. Nevertheless, this case would be interesting since the same collection of substructures could also determine similar pharmacological and distribution profiles.

In the current study, the performances of different 2D fingerprints analyzing the molecular structure from diverse points of view were described. However, alternative methods using other types of fingerprints or molecular descriptors could still be explored [44], [45]. Another possibility for further study is the construction of similar models taking into account the 3D most stable conformation in drugs [46]. Nevertheless, although 2D methods are considered more limited than 3D methods, they still present good results and it is possible to avoid complex steps, such as the selection of bioactive drug conformations, calculation of the 3D most stable conformers and superimposition of the final structures to compare their similarity.

### Conclusions

The combination of EHR analysis and structural similarity data led to an improved prioritization of drug candidates related to the adverse event *pancreatitis*. A set of drugs was selected using the combination of both techniques to further study their possible causal relationship to *pancreatitis*. The results obtained in this study are in accordance with a previous publication analyzing the adverse event *rhabdomyolysis* [10]. Structural similarity analysis could be used as a useful tool to analyze and rationalize data extracted from pharmacovigilance databases. The implementation of molecular structure data can facilitate adverse drug event detection.

## Supporting Information

Table S1 **Reference standard of 253 drugs reported to cause the ADE** ***pancreatitis*** **.**
(XLSX)

Table S2 **Drug candidates selected in EHR. Similarity against the** ***pancreatitis*** **reference standard dataset is provided through Tanimoto coeficcient (TC) and different fingerprints (TGT, GpiDAPH3, TGD, MACCS).** Available bibliographic information relating the drug with *pancreatitis* is provided.
(XLSX)
